# Structural brain abnormalities in Pallister-Killian syndrome: a neuroimaging study of 31 children

**DOI:** 10.1186/s13023-024-03065-5

**Published:** 2024-03-08

**Authors:** Anna Fetta, Francesco Toni, Ilaria Pettenuzzo, Emilia Ricci, Alessandro Rocca, Caterina Gambi, Luca Soliani, Veronica Di Pisa, Silvia Martini, Giacomo Sperti, Valeria Cagnazzo, Patrizia Accorsi, Emanuele Bartolini, Domenica Battaglia, Pia Bernardo, Maria Paola Canevini, Anna Rita Ferrari, Lucio Giordano, Chiara Locatelli, Margherita Mancardi, Alessandro Orsini, Tommaso Pippucci, Dario Pruna, Anna Rosati, Agnese Suppiej, Sara Tagliani, Alessandro Vaisfeld, Aglaia Vignoli, Kosuke Izumi, Ian Krantz, Duccio Maria Cordelli

**Affiliations:** 1 IRCCS Istituto delle Scienze Neurologiche di Bologna, UOC di Neuropsichiatria dell’Età Pediatrica, Bologna, Italy; 2 Dipartimento di Scienze Mediche e Chirurgiche (DIMEC), Università di Bologna, Bologna, Italy; 3 IRCCS Istituto delle Scienze Neurologiche di Bologna, Programma di Neuroradiologia con Tecniche ad elevata complessità– PNTEC, Bologna, Italy; 4 Epilepsy Center, Childhood and Adolescence Neuropsychiatry Unit, ASST Santi Paolo e Carlo, San Paolo Hospital, 20142 Milan, Italy; 5 UO di Pediatria d’Urgenza, IRCCS Policlinico Sant’Orsola, Bologna, Italy; 6 Neonatal Intensive Care Unit, IRCCS AOUBO, Bologna, Italy; 7 Scuola di Specializzazione in Pediatria - Alma Mater Studiorum, Università di Bologna, Bologna, Italy; 8 Child Neuropsychiatric Division, Spedali Civili, Brescia, Italy; 9 Department of Developmental Neuroscience, IRCCS Stella Maris Foundation, 56128 Pisa, Italy; 10 Pediatric Neurology, Department of Woman and Child Health and Public Health, Child Health Area, Catholic University UCSC, Rome, Italy; 11 Department of Neurosciences, Pediatric Psychiatry and Neurology Unit, Santobono-Pausilipon Children’s Hospital, Naples, Italy; 12 Unit of Child Neuropsychiatry, IRCCS Istituto Giannina Gaslini, Epicare Network for Rare Disease, Genoa, Italy; 13 Pediatric Neurology, Pediatric University Department, Azienda Ospedaliera Universitaria Pisana, University of Pisa, Pisa, Italy; 14 U.O. Genetica Medica, IRCCS Azienda Ospedaliero-Universitaria di Bologna Policlinico S Orsola, Bologna, Emilia- Romagna, Italy; 15 Department of Pediatric Neurology and Epileptology, Pediatric Depatment, ARNAS Brotzu, Cagliari, Italy; 16 Neuroscience Department, Children’s Hospital Anna Meyer, University of Florence, Viale Gaetano Pieraccini, 24, 50139 Firenze, Italy; 17 Department of Medical Sciences, Pediatric Section, University Hospital of Ferrara, Ferrara, Italy; 18 Child Neuropsychiatry Unit, Department of Health Sciences, ASSTGrande Ospedale Metropolitano, Niguarda, Milano, Italy; 19 Division of Genetics and Metabolism, Department of Pediatrics, University of Texas Southwestern Medical Center, 5323 Harry Hines Blvd., 75390 Dallas, TX USA; 20 Divisions of Human Genetics, Children’s Hospital of Philadelphia, Philadelphia, PA USA

## Abstract

**Background:**

Pallister-Killian syndrome (PKS) is a rare genetic disorder caused by mosaic tetrasomy of 12p with wide neurological involvement. Intellectual disability, developmental delay, behavioral problems, epilepsy, sleep disturbances, and brain malformations have been described in most individuals, with a broad phenotypic spectrum. This observational study, conducted through brain MRI scan analysis on a cohort of patients with genetically confirmed PKS, aims to systematically investigate the neuroradiological features of this syndrome and identify the possible existence of a typical pattern. Moreover, a literature review differentiating the different types of neuroimaging data was conducted for comparison with our population.

**Results:**

Thirty-one individuals were enrolled (17 females/14 males; age range 0.1–17.5 years old at first MRI). An experienced pediatric neuroradiologist reviewed brain MRIs, blindly to clinical data. Brain abnormalities were observed in all but one individual (compared to the 34% frequency found in the literature review). Corpus callosum abnormalities were found in 20/30 (67%) patients: 6 had callosal hypoplasia; 8 had global hypoplasia with hypoplastic splenium; 4 had only hypoplastic splenium; and 2 had a thin corpus callosum. Cerebral hypoplasia/atrophy was found in 23/31 (74%) and ventriculomegaly in 20/31 (65%). Other frequent features were the enlargement of the cisterna magna in 15/30 (50%) and polymicrogyria in 14/29 (48%). Conversely, the frequency of the latter was found to be 4% from the literature review. Notably, in our population, polymicrogyria was in the perisylvian area in all 14 cases, and it was bilateral in 10/14.

**Conclusions:**

Brain abnormalities are very common in PKS and occur much more frequently than previously reported. Bilateral perisylvian polymicrogyria was a main aspect of our population. Our findings provide an additional tool for early diagnosis.Further studies to investigate the possible correlations with both genotype and phenotype may help to define the etiopathogenesis of the neurologic phenotype of this syndrome.

## Background

Pallister-Killian syndrome (PKS; OMIM 601,803) is a sporadic and rare genetic developmental disorder with multisystemic involvement caused by the presence of a supernumerary isochromosome 12p– i(12)(p10) that determines 12p tetrasomy, with a mosaic distribution in different cell lines and tissues [1–4]. In 2012, Izumi et al. hypothesized the presence of a minimal critical region within 12p13.31 responsible for the core phenotype of PKS [5]. To date, more than 200 individuals have been described in the literature [4]. 

Given the mosaicism, clinical manifestations are various, including characteristic craniofacial dysmorphism, pigmentary skin differences, and congenital anomalies (mainly brain abnormalities, heart defects, and visceral and limb anomalies) [1–4]. The neurological phenotype is a key aspect of PKS: most individuals present different degrees of intellectual disability, developmental delay, behavioral problems, epilepsy (from epileptic spasms to focal to generalized seizures, including myoclonic epilepsy with low-frequency photosensitivity), sleep disturbances, and brain malformations, which are variously combined and intrinsically linked [6–13]. 

Structural brain anomalies (SBAs) have been reported in up to 73% of PKS individuals. In a retrospective analysis of 114 pregnancies associated with a literature review of 86 published cases, Salzano et al. identified some major second-trimester ultrasound markers to raise the suspicion of PKS, including cerebral ventriculomegaly [14]. Other authors reported brain ultrasound findings such as periventricular cysts, corpus callosum (CC) thinning, mega cisternamagna, and cerebellar anomalies up to Dandy-Walker malformation. Recently, Barkovich et al. reported neuroimaging findings in 42 PKS individuals collected from the literature, and subsequently, Poulton et al. surveyed 150 published reports to identify neuroradiological findings of 93 PKS individuals. That literature review revealed a wide spectrum of SBAs: cerebral brain volume loss up to cerebral atrophy, malformations of cortical development such as polymicrogyria (PMG), CC dysgenesis, ventricular abnormalities, and, less often, white matter (WM) anomalies. Nevertheless, it was not possible through that revision to differentiate between magnetic resonance imaging (MRI),computer tomography (CT), ultrasound (US), or anatomopathological findings [8, 11]. Moreover, to date, there is no population study on SBAs in PKS.

Therefore, our study aims to further characterize the neuroradiological features of PKS in a large population looking for any potential recurrent pattern; furthermore, to conduct a new literature review distinguishing between the different types of neuroimaging data to be able to compare it with our population; and finally, to make an initial attempt to relate the neuroradiological findings to the clinical and demographic features.

## Methods

### Population and data collection

From January 2018 to December 2022, a cohort of individuals with genetically confirmed PKS and available brain MRI data was recruited through the collaborative efforts of the contributing authors. All cases were sporadic.

All patients were clinically evaluated at their referral center. Clinical-demographic information and at least one MRI were collected for each patient. The history of epilepsy, the developmental level,and evidence of severe vision impairment at the last evaluation were considered. Moreover, available Sleep Disturbance Scale for Children (SDSC) scores were collected [15]. Personal information was elaborated into an anonymized form through the attribution of a unique progressive code to each patient. Different clinical aspects from subsets of this cohort have been previously evaluated [9, 10, 12, 13]. 

MRI exams included at least a two or three-dimensional (2D or 3D) sagittal and/or axial T1-weighted (T1-w) sequence, with multiplanar (MPR) reconstruction, 2D axial or coronal T2-weighted image (T2-w), and 2D axial or 3D sagittal with MPR fluid-attenuated inversion recovery (FLAIR) sequence.

A pediatric neuroradiologist (FT) reviewed and scored all MRIs, blindly to clinical features; PKS diagnosis, sex, and age at MRI were unblinded. SBAs were evaluated for the presence of signal abnormalities and/or abnormal metrics. Brain metrics were obtained by using the Carestream PACS visualization Workstation. When more than one MRI was available, all were analyzed. When differences were present between the two investigations, they were reported.

Ventricular system dilatation, mainly supratentorial, was evaluated using a qualitative severity scale (normal, mild, moderate, severe). Temporal horn enlargement and atrial widening were noted, both indirect signs of reduced posterior CC commissure. An analogous qualitative scale was applied to the brain volume reduction according to the degree of cerebral sulci enlargement and subarachnoid spaces (SAS) dilatation. The distinction between sulci and SAS was added in an effort to better characterize glymphatic system function. Brain hypoplasia and atrophy represent two different forms of reduced brain volume. Nevertheless, especially if follow-up studies are lacking, it is challenging to discern if reduced brain volume is due to the presence of neurodegeneration leading to progressive atrophy, if it is related to an alteration of physiological microstructural development causing hypoplasia, or if both mechanisms are involved [16]. Thus, cerebral volume reduction was identified as “brain hypoplasia/atrophy”.

The qualitative evaluation of CC was performed by classifying it as normal, thin and bowed, globally hypoplastic, globally hypoplastic with prominent involvement of the splenium, or normal except for a hypoplastic splenium. Moreover, a quantitative evaluation was achieved. According to the Garel et al. biometry model for CC [17], we obtained the following biometric parameters: the anteroposterior diameter (APD) of the CC, measuring the distance between the anterior aspect of the genu and the posterior aspect of the splenium; the true length of the CC (LCC), measuring the curvilinear distance between the rostrum and the splenium at the mid-thickness of the CC; the frontal-occipital diameter (FOD), measuring the distance between the extreme points of the frontal and occipital lobes; the distance splenium/tegmentum (S/T), measuring the distance at the level of the fastigium, between a line drawn along the dorsal surface of the brain stem and another line parallel to the first one and passing through the level of the most posterior point of the splenium; and the thickness of the CC, measured at the level of the genu (GT), the body (BT), the isthmus (IT), and the splenium (ST). The obtained values were then compared with the reference population and categorized as “< 3rd percentile,” “between 3rd and 15th”, “between 15th and 85th”, “between 85th and 97th”, and “>97th" [17].

Parenchymal abnormalities of cerebral gray and white matter and posterior fossa structures were qualitatively evaluated and described. Moreover, the Jandeaux biometry model [18] was used for the cerebellum, pons, and midbrain: 2D measurements were manually performed on a sagittal section passing through the midsagittal plane and were defined as follows: the height of the vermis (H-V) was the largest craniocaudal diameter of the vermis; the anterior-posterior diameter of the vermis (APD-V) was the largest anterior-posterior diameter of the vermis passing through the tip of the V4; the anterior-posterior diameter of the midbrain-pons junction (APD-MP) was measured perpendicular to the major axis of the brain stem passing through the midbrain-pons junction; and the anterior-posterior mid pons diameter (APD-P) was measured perpendicular to the major axis of the brain stem passing through the middle of the pons. The values obtained were then compared with the reference population and categorized as “< 3rd percentile,” “between 3rd and 97th”, and “>97th” [18]. 

### Literature review

A review of the literature was performed by searching PubMed and Google Scholar and by additional hand searches through reference lists. The terms “Pallister-Killian syndrome” or “PKS” AND “neuroimaging” or “intracranial scan” or “brain MRI” were used for the research. The results were filtered for case reports, case series, and preview studies. The identified reports were screened manually to identify individuals with PKS fulfilling the inclusion criteria (i.e., sufficient neuroradiological or anatomopathological data available). Only papers published in English were reviewed.

### Statistical analysis

The clinical and neuroradiological data were analyzed through descriptive statistics; continuous variables are expressed as medians with interquartile ranges (IQRs), and categorical variables are expressed as absolute numbers and percentages. Comparisons between the two groups were made using the U Mann‒Whitney test for independent datasets. For categorical variables, we used the chi-square test or Fisher’s test with Bonferroni correction post hoc. All analyses were conducted in JASP (Version 0.17.1) Microsoft version, and *p* ≤ 0.05 from 2-sided tests was considered statistically significant.

For the statistical correlation analysis, 5 categorical neuroradiological variables were identified (CC, WM, and pineal gland abnormalities, brain hypoplasia/atrophy, and malformations of cortical development -MCD-) and correlated with sex, age, and clinical features (developmental delay, vision impairment, epilepsy, and SDSC scores).

Moreover, for the definition of brain hypoplasia/atrophy, the degree of SAS and sulci enlargement was correlated with the ventricles dilatation.

Two age groups were identified through the median of the ages at first MRI and defined as follows: “younger” group composed of individuals aged < of the median; “older” group composed of individuals aged ≥ of the median.

### Ethics

This study was conducted in conformity with the principles and regulations of the Helsinki Declaration. Informed consent to participate was obtained from the infants’ parents/legal guardians. The local ethics committee approved the study.

## Results

### Patient characteristics

Our cohort was composed of 31 individuals (17 females/14 males); the median age at the first brain MRI was 1.6 years old (IQR 3.8, range 0.1–17.5).

We evaluated 37 MRIs. For 25 individuals, a single brain MRI examination was performed, while for 6, two MRI examinations were performed with a median time between the scans of 1.9 years: two within 5 months, one after 13 months, two between 2.6 years and 3.3 years, and one 12.7 years apart.

Demographic and clinical information, as well as genetic and molecular studies are summarized in Table 1.

**Table 1:** Clinical and Demographic Characteristics

Code	Sex	Genetics	n° of MRIs	Age at 1st MRI (years)	Time between MRIs (years)	Age at last clinical evaluation (years)	Seizures	Severe Vision Impairment	Developmental delay	SDSC score
Case 1	f	n/a	1	0.1		1.2	n	y	severe	n/a
Case 2	f	47, XX, i(12)(p10) [1]/46, XX,[99] (karyotype on blood)	1	0.1		3	y	n	moderate	50
Case 3	m	47,XY, i(12)(p10) [19]/46,XY [16] (karyotype on fibroblasts from hyperchromic skin biopsy) arr[GRCh37] 12p13.33p11.1 (191619_34853011)x2-4 (SNP array on hyperchromic skin biopsy)	2	0.3	2.6	3	y	n	moderate	54
Case 4	f	arr12p13.33p11.1 (Array-CGH on unknown sample)	1	0.3		7.2	y	n/a	n/a	n/a
Case 5	f	arr[GRCh37] 12p13.33p11.1 (191619_34826574)x2-4 (Array-CGH on unknown sample)	2	0.4	0.4	2.4	n	n	severe	70
Case 6	f	arr[hg19]12p13.33p11.1 (0-43257397 × 3, 34,294,005 × 2) (Array-CGH on unknown sample)	1	0.4		1.4	n	y	severe	n/a
Case 7	m	47, XY, +i(12)(p10) [20]/46, XY [4] (Karyotype on fibroblasts)	1	0.8		2.5	y	y	severe	90
Case 8	f	arr12pterp11.1 (163,593 − 34,398,316)x2-4 (Array-CGH on blood)	1	0.8		10.8	y	n	mild	45
Case 9	f	arr [hg19] 12p13.33p11.1 (163, 679 − 34,760,977) x2 3 (Array-CGH on blood)	2	0.9	0.4	1.5	n	n	severe	64
Case 10	f	ish12(D1221 × 3,wep12 × 3,pcp12p11.2p13 × 4) [21]/12(D12Z1 × 2,wep12 × 2, pep12p11.2p13 × 2) [22] (FISH on fibroblasts)	2	0.9	3.3	3.7	y	n/a	moderate	n/a
Case 11	m	47, XY, i(12p) [7]/46, XY [23] (karyotype on fibroblasts)	2	1	1.2	6.4	y	y	severe	99
Case 12	f	arr12p13.33p11.1 (Array-CGH on buccal epithelial cells)	1	1		6.3	n	y	severe	62
Case 13	m	n/a	1	1.2		7.5	y	y	n/a	n/a
Case 14	m	Mos 47, XY arr[hg19] 12p13.33p11.1(148,375 − 34,760,977)x3 (karyotype on blood)	1	1.4		7.4	y	n	severe	n/a
Case 15	m	47,XY + i(12p) [22],46,XY [5].ishi(12p)(cp12) (karyotype on fibroblasts)	1	1.5		1.4	y	y	severe	n/a
Case 16	m	n/a	1	1.6		2.7	y	y	severe	n/a
Case 17	f	47,XX,+i(12)(p10) [6]/46,XX [4] (karyotype on blood); arr[GRCh37)12p13.33p11.1(194249_34398316)x3-4 (Array-CGH on blood)	1	2		4.3	n	n	mild	n/a
Case 18	f	47,XX, +i(12p)(p10) [24]/46,XX [3] (karyotype on fibroblasts)	1	2.4		2.4	n	n	mild	62
Case 19	m	47, XY, i(12)(p10)/46, XY (karyotype on fibroblasts)	1	2.7		16.4	y	y	severe	n/a
Case 20	m	arr12p13.33p11.2 (Array-CGH on unknown sample)	1	3		9	y	n/a	severe	n/a
Case 21	f	Ish 12p13(TELx2) [25]/12p13 (TEL x 3) [26] 12p13(TELx4 [24] (FISH on buccal epithelial cells)	1	3.7		4.5	y	n	mild	n/a
Case 22	f	47, XX, i(12)(p10) [2]/46, XX [98] (karyotype on amniocentesis)	1	4.1		11.8	n	y	severe	51
Case 23	f	arr [hg19] 12p13.33p11.1 (163, 618 − 34,756,180) x2-3 (Array-CGH on blood)	1	4.5		10.2	y	y	moderate	100
Case 24	f	46, XX [21],47XX + i(12p) [22] (karyotype on fibroblasts)	1	4.9	12.7	17.6	y	y	severe	68
Case 25	f	Ish 12p13 (TELx4) [27]/(TELX3) [28]/(TELX2) [29] (FISH on buccal epithelial cells)	1	4.9		5	y	y	severe	n/a
Case 26	m	n/a	1	7		18.2	y	y	severe	n/a
Case 27	m	12p13.33p11.1 (Array-CGH on unknown sample)	1	9.1		11.7	y	n	mild	n/a
Case 28	m	47,XY, i(12)(p10) (karyotype on blood)	1	11.4		17.4	y	y	severe	62
Case 29	f	47, XX, i(12p) (karyotype on blood)	1	11.6		11.6	y	n	moderate	69
Case 30	m	47, XY, i(12)(p10)/46, XY (karyotype on buccal epithelial cells)	1	13.1		14.4	n	n	severe	62
Case 31	m	47, XX, i(12p) (karyotype on fibroblasts)	1	17.5		17.6	y	n/a	severe	n/a

Legend: n° =number; MRI = magnetic resonance imaging; SDSC = Sleep Disturbance Scale for Children; f = female; m = male; y = yes; n = no; n/a = not available. Clinical data (seizures, severe vision impairment, developmental delay, SDSC score) refer to the latest available evaluation

At last clinical evaluation, epilepsy occurred in 22 of 31 patients; all presented developmental delay/intellectual disability of various degrees: 19 severe, 5 moderate, 5 mild, and 2 unknown levels of developmental delay. Fifteen patients had severe vision impairment, whereas 12 did not; this information was not available for 4.

### Neuroradiological findings

#### Brain trophism and ventricular system

Sulci enlargement evaluation, as the main marker of brain trophism, was performed in all 31 individuals. Brain hypoplasia/atrophy was found in 23 (74%) cases, classifiable as mild in 8, moderate in 12, and severe in 3. Additionally, SAS enlargement was present in 23 cases (7 mild, 11 moderate, and 5 severe). In case 3 and case 10, this latter item improved at the second MRI performed 2.6 years and 3.3 years later, respectively (case 3 went from mild to no enlargement, and case 10 went from moderate to mild enlargement). In the remaining 4 cases, the finding was stable at the second MRI.

**Fig. 1:**
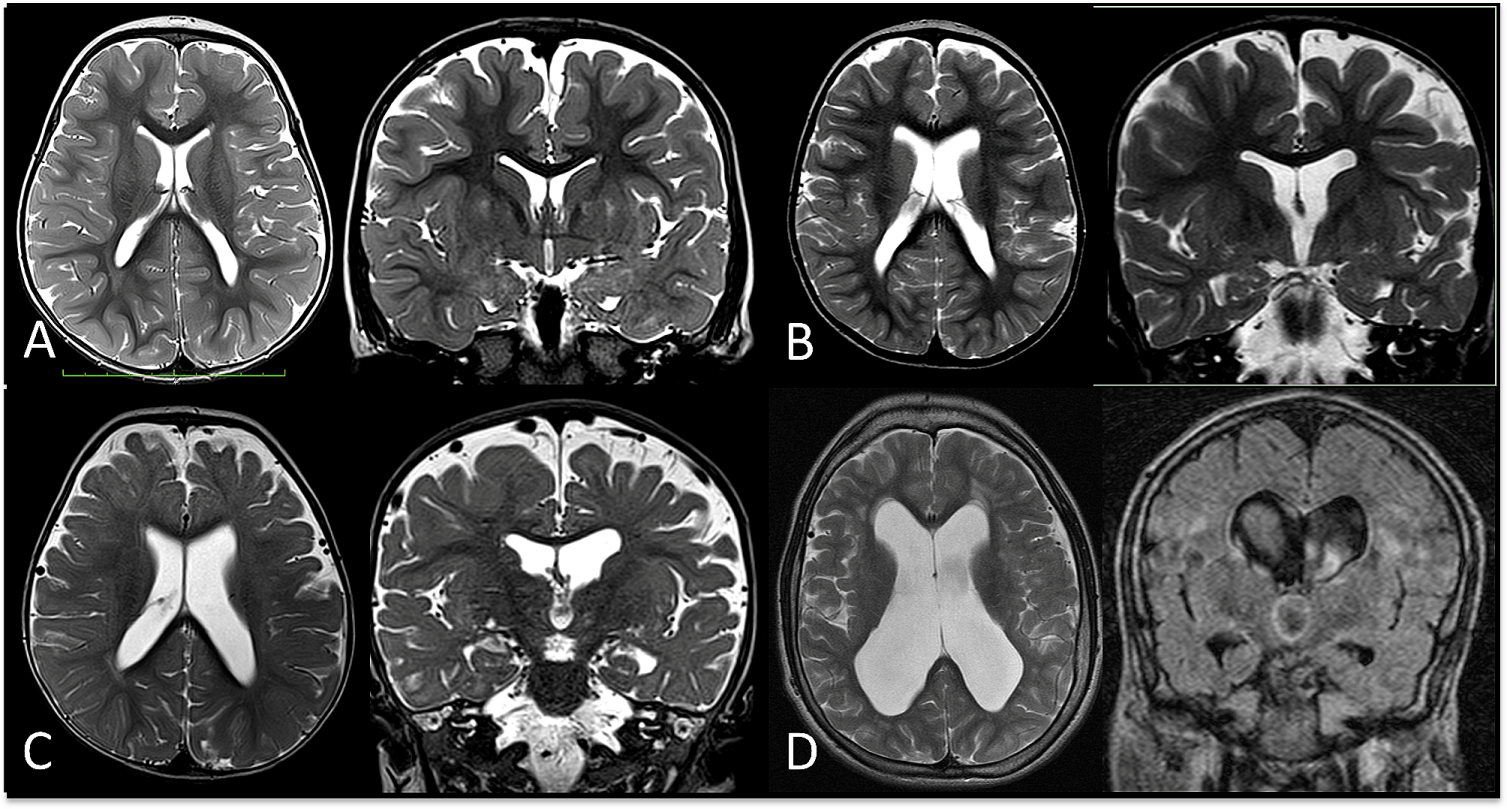
**Brain Trophism and Ventricular System. A**, Regular volume of the ventricular system with a slight widening of the peri-cerebral subarachnoid spaces (Case 18, 2 years old); **B**, mild enlargement of the lateral ventricles and of the peri-cerebral subarachnoid spaces (Case 22, 4 years old); **C**, more prominent ventriculomegaly and dilatation of the peri-cerebral subarachnoid spaces (Case 11, 1 year old); **D**, communicating hydrocephalus, with signs of hyperdynamic flow (flow-related artifact on coronal FLAIR on the right) (Case 31, 17 years old)

Ventricular enlargement was found in 20 (65%) patients: the degree of dilatation was mild in 10 patients, moderate in 7 patients, and severe in 3 patients. Among the latter, it resulted from communicating hydrocephalus in 2 (Fig. 1).

Regarding the morphology of the ventricles, 21 patients had an enlargement of the temporal horns, of which 6 were in the context of normal-sized ventricles, and 15 were concurrent with enlarged ventricles (7 slightly, 7 moderately, and 1 severely). Atrial widening was present in 21 cases (18 together with temporal horn enlargement), of which 6 were in the context of normal ventricular volume.

#### The severity of ventricular enlargement was significantly correlated with the severity of sulci (*p* = 0.004) and SAS (*p* = 0.004) enlargement. Corpus Callosum

**Fig. 2:**
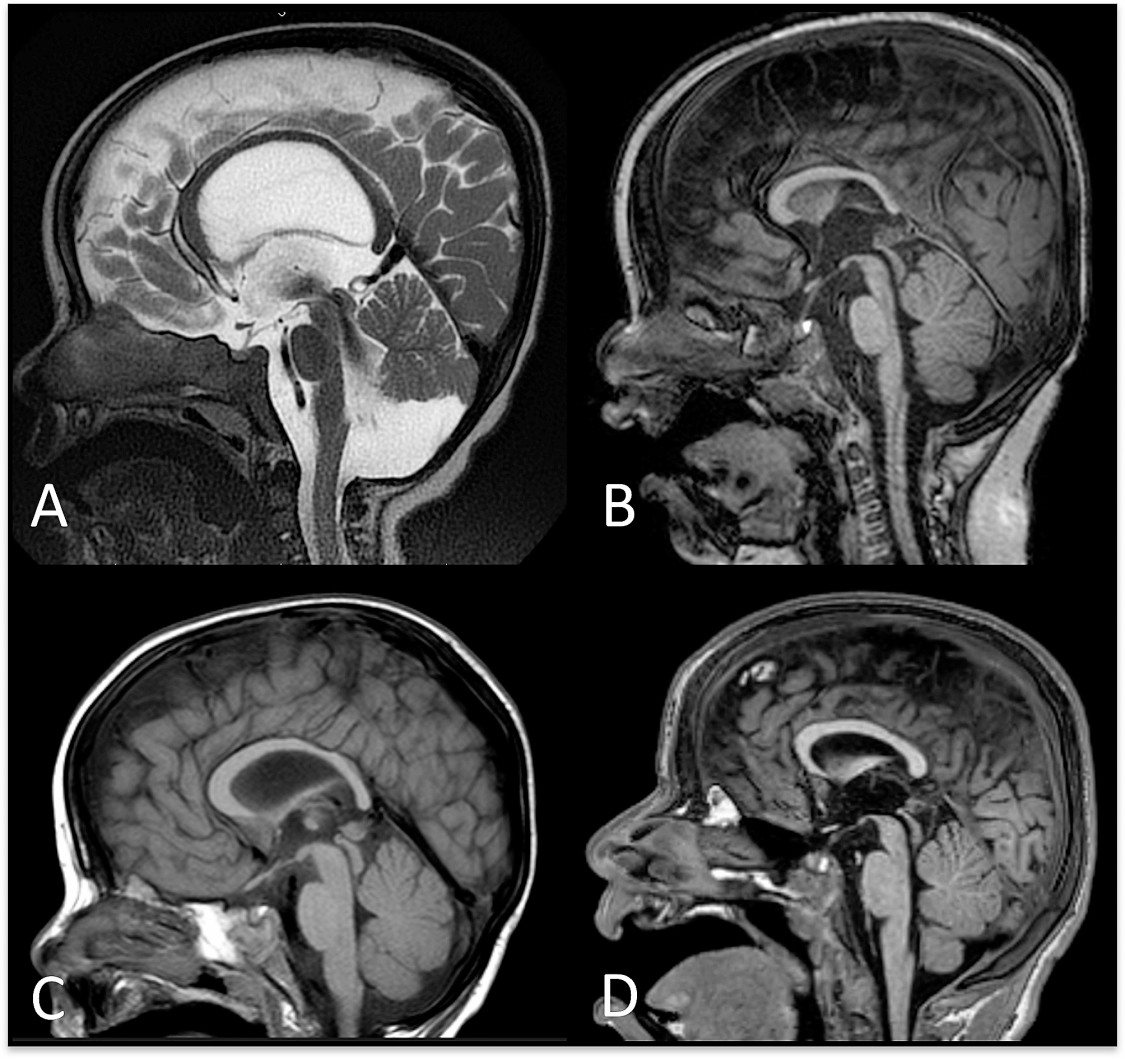
**Corpus Callosum Morphology.A**, Thinning and bowing due to communicating hydrocephalus (Case 9). **B**, hypoplasia of the corpus callosum, more evident at the splenium (Case 16); **C**, hypoplasia of the splenium with regular biometry of the other component of the corpus callosum (Case 29); **D**, global and harmonic hypoplasia of the corpus callosum (Case 31)

CC was qualitatively evaluated in 30/31 cases, and abnormalities were found in 20/30 (67%). The qualitative assessment showed the CC to be thin and bowed in 2 patients (those who had hydrocephalus), globally hypoplastic in 6, and globally hypoplastic with prominent involvement of the splenium in 8; 4 cases had a CC of normal volume except for a thinned conformation of the splenium (Fig. 2).

**Fig. 3:**
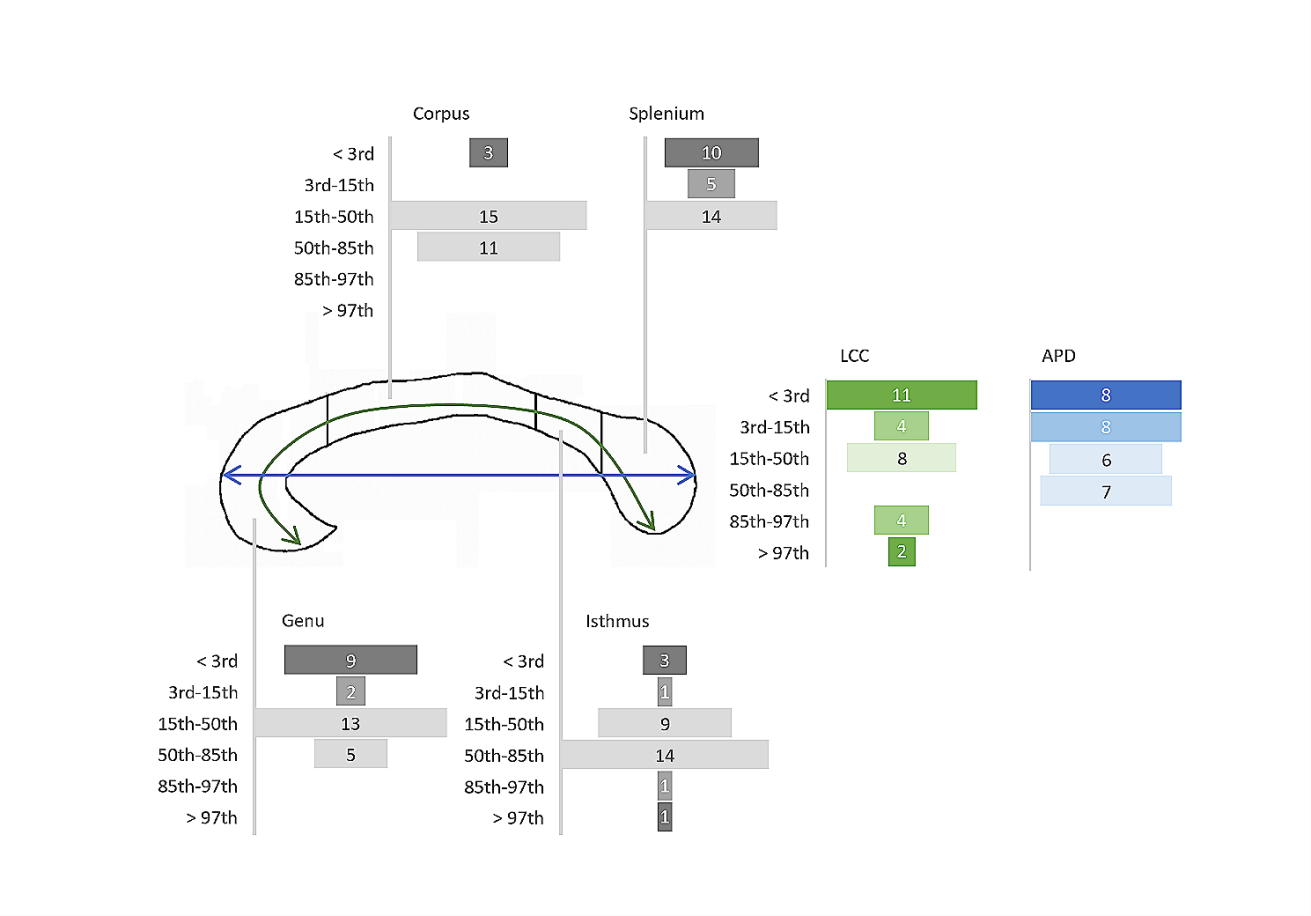
**Corpus Callosum Biometry.** The figure shows the distribution of biometric data in the analyzed population with respect to age reference percentiles. LCC: length of corpus callosum (curvilinear distance between the rostrum and the splenium at the mid-thickness of the corpus callosum); APD: anteroposterior diameter (distance between the anterior aspect of the genu and the posterior aspect of the splenium)

For the biometric evaluation, a population of 29 cases was included. Extended results are reported in Fig. 3.

#### Cerebral parenchymal abnormalities

Cerebral parenchymal signal alterations were found in 6/31 (19%) individuals. One of them had temporal-parietal-insular injury as a result of a perinatal stroke in the right middle cerebral artery territory (Case 26), one had deep WM and periventricular gliosis of probable vascular etiology (Case 31), one showed abnormal myelin development (Case 13), and three had lesions suggestive of perinatal hypoxic-ischemic lesions (focal gliosis of the Rolandic deep WM in Case 11, periventricular leukomalacia (PVL) with unilateral frontal-parietal-insular ulegyric cortex in Case 17, and PVL, left parietal-insular ischemic change and bilateral posterior limb of internal capsule/thalamic gliosis in Case 25). Two of them (Cases 17 and 25) had a clear history of perinatal injury requiring assistance in the neonatal intensive care unit.

#### Cerebellum and brainstem

Posterior cranial fossa abnormalities were found in 16/30 (53%) cases, without differences in relation to age and sex. Fifteen patients had isolated enlargement of the cisterna magna, of which 2 were associated with counterclockwise rotation of the cerebellar vermis. One subject presented only cerebellar vermis rotation.

Biometry of the vermis, pons, and cerebellum was performed in 28/31 patients: all 28 presented values in the normal range for the vermis; 5 (20%) had an APD-P below the 3rd percentile, while the remaining 20 (80%) had values in the normal range; 4 (16%) had an APD-MP below the 3rd percentile, while the remaining 21 (84%) were normal.

#### Malformations of cortical development

**Fig. 4:**
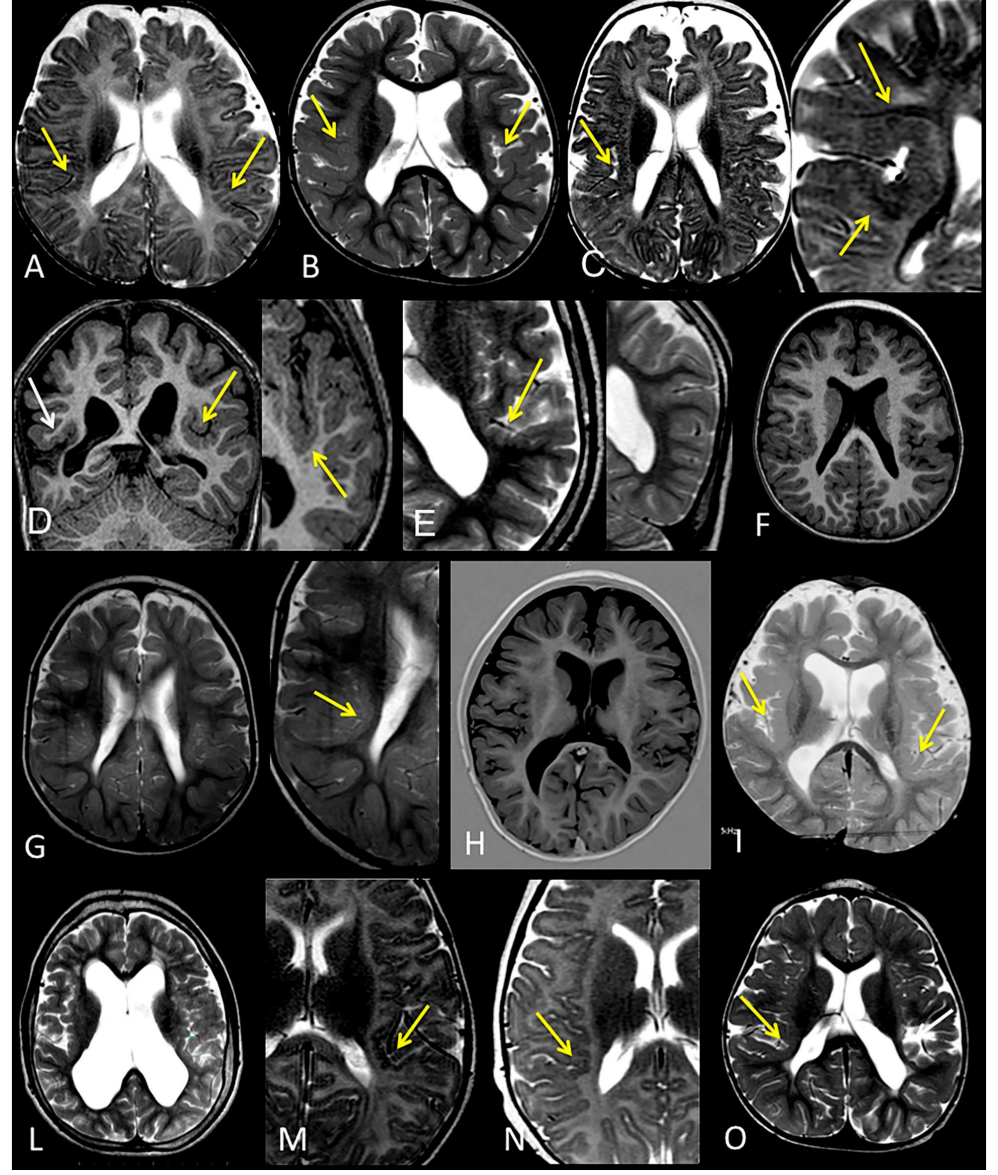
Perisylvian Polymicrogyria. **A**, Bilateral perisylvian polymicrogyria (Case 6); **B**, bilateral perisylvian polymicrogyria (Case 2); **C**, right perisylvian polymicrogyria (Case 7); **D**, left perisylvian polymicrogyria (yellow arrow) and right ischemic clastic perisylvian lesion (white arrow) (Case 26); **E**, left perisylvian polymicrogyria (yellow arrow) and monomorphic gyri related to dysgiric cortex in the left parietal-temporal-occipital lobe (Case 28); **F**, bilateral perisylvian polymicrogyria (Case 22); **G**, bilateral perisylvian polymicrogyria (Case 16); **H**, bilateral polymicrogyria (Case 24), more evident on the left side; **I**, bilateral polymicrogyria (Case 19); **L**, bilateral polymicrogyria (Case 31); **M**, left perisylvian polymicrogyria (yellow arrow) (Case 5); **N**, right perisylvian polymicrogyria (yellow arrow) (Case 3); **O**, right perisylvian polymicrogyria (yellow arrow) and left ischemic clastic perisylvian lesion (white arrow) (Case 17)

MCD were found in 14 (48%) out of 29 evaluated patients (2 individuals were discarded due to the low resolution of the MRI scans), with no difference in relation to sex. All had perisylvian PMG, of which 10 were bilateral (Fig. 4); in two out of the four cases with unilateral PMG (Cases 3, 4, 17, and 26), perisylvian PMG co-occurred with a contralateral ischemic clastic lesion in the same area (Cases 17 and 26) (Fig. 4D and O).

#### Pineal gland

The pineal gland was evaluated in 28/31 individuals, showing cystic appearance in 18/28 (64%). It was only enlarged in 3, microcystic in 6, cystic in 8, and both cystic and enlarged in 1. One patient developed a cystic tumor (Case 17).

#### Demographic and clinical correlations

No significant correlations were found in relation to sex, age, or the clinical parameters examined (developmental delay, vision impairment, epilepsy, or SDSC scores).

### Literature review

A total of 65 articles (including 140 individuals with PKS) were identified by literature review; each of them was screened for data about structural brain features. Six articles (21 cases) were excluded because insufficient data were gathered in the text.

**Fig. 5:**
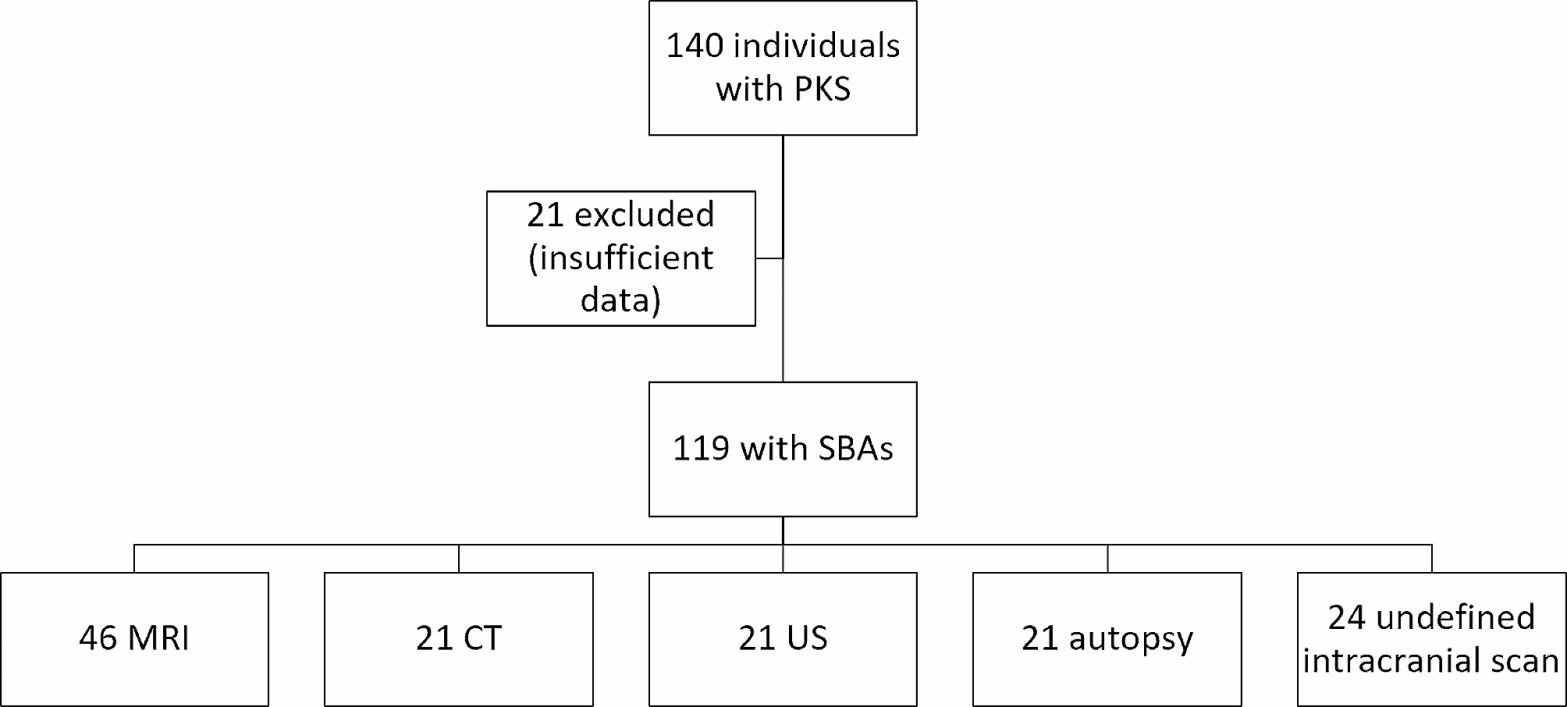
**Literature Review Flowchart.** PKS: Pallister Killian syndrome; SBAs: structural brain abnormalities; MRI: magnetic resonance imaging; CT: computer tomography; US: ultrasound

Structural brain information was reported in 119 individuals with PKS, each of whom underwent one or more different intracranial examinations (Fig. 5). Postmortem examination information was included when providing comparable data (e.g., ventriculomegaly, hydrocephaly, cerebellar defects, cortical malformation). Data about “intracranial scan” (without further details) were described in 24 individuals.

The main SBAs reported in the literature and their respective frequencies, as well as a comparison with the frequencies found in our population, are shown in Table 2.

**Table 2:** Literature Review

Structural Brain Abnormalities	Literature data						Our sample data
	MRI (frequency) [2, 13, 30–46]	CT [11, 24, 23, 22, 36, 41, 42, 47–54]	US [31, 23, 22, 55–66]	Undetermined scan [3, 23]	Autopsy [57, 59–61, 63, 64, 67–75]	Total reported cases (frequency)	Total reported cases (frequency)
Reduced brain trophism	14 (30%)	8	2	-	-	21 (18%)	23 (74%)
Ventricular abnormalities	16 (35%)	4	9	3	7	31(26%)	20 (65%)
Corpus callosum	11 (24%)	1	-	5	2	17 (14%)	18 (58%)
White matter abnormalities	3 (7%)		1	2	2	9 (8%)	7 (22%)
Cerebellar structural defects	1 (2%)	2	1	-	5	8 (7%)	3 (10%)
Hydrocephalus/hygromas	1 (2%)	3	-	-	3	7 (6%)	2 (6%)
Polymicrogyria	4 (9%)	1	-	-	2	6 (5%)	15 (48%)
Pachygyria	3 (7%)	-	-	-	-	3 (3%)	-
Cisterna magna	-	-	3	-	1	3 (3%)	15 (48%)
Pineal gland	1 (2%)	-	-	-	-	1 (1%)	18 (58%)
Calcification	-	3	2	-	1	6 (5%)	-
Others*	1 (2%)	2	1	-	3	8 (7%)	-
No abnormal features	13 (28%)	9	3	12	3	40 (34%)	1 (3%)
Total neuroimaging scans	46 (100%)	21 (100%)	21 (100%)	24 (100%)	21 (100%)	119 (100%)	31(100%)

Legend: MRI = magnetic resonance imaging; CT = computer tomography; US = ultrasound; *=olfactory nerve, germinal matrix hemorrhage and ischemic thrombi in the subependymal veins, atrophic brainstem, small cava septi pellucidi

## Discussion

In this study, we conducted the first systematic evaluation of brain MRI scans from a large population of individuals with PKS. Moreover, we conducted a review of the available data from the literature, comparing them to our population.

We found that only one of the enrolled patients showed no brain MRI abnormalities (compared with 28% of the MRI and 34% of the total cases described in the literature), suggesting a greater structural brain involvement than previously reported. This could be due to a more accurate and targeted analysis of brain MRI scans since we carefully checked for CNS abnormalities; literature data were mostly from case reports and exams other than MRIs, not specifically focusing on the neuroradiological aspect. Both our results and the major frequency of SBAs in the subgroup of the literature studied with MRI suggest that this exam can be considered the gold standard in the neuroradiological study of these patients.

The 12p duplicated region appears to be rich in genes involved in neuropathological features, as Poulton et al. reported in their review, identifying an overlap between the minimal critical region spanning 12p13.33 to 12p13.2 for cerebral atrophy and from 12p13.33 to 12p12.3 for CC anomalies, both including dozens of genes and extending the previous findings of Izumi et al. about the minimal critical region responsible for PKS [5, 8]. 

The most common brain abnormality in our population was brain volume loss (identified as hypoplasia/atrophy and defined as decreased parenchymal volume associated with ventricular, sulcal, and cisternal space enlargement), which was more than two times more frequent than previously reported [8]. As previously mentioned, it is challenging to discern if reduced brain volume is due to progressive atrophy, to microstructural underdevelopment causing hypoplasia, or if both mechanisms are involved [16]. Most of our population had MRIs at a noticeably early age, and there were few examinations of older children. Moreover, most of the follow-up examinations were remarkably close in time, not allowing us to assess whether there was a progression of atrophy.

Hypoplasia/atrophy affected brain hemispheres rather than other structures and was often associated with ventriculomegaly [11]. In our series, ventricular dilatation was indeed significantly correlated with the severity of sulci and SAS enlargement. Nevertheless, we decided to define the hypoplasia/atrophy item relying mainly on the degree of cerebral sulcal widening, since both the SAS and the ventricular system could be more susceptible to variation related to glymphatic system alteration or other pathological cerebrospinal fluid mechanism abnormalities. This could be hypothesized and exemplified by two patients who had hydrocephalus associated with severe ventriculomegaly or two cases with SAS enlargement, which improved at the second MRI.

Genetically, the abovementioned 12p13.33 to 12p13.2 region, known to be involved in the context of other syndromes, seems to be responsible for this alteration [5, 76–77, 16]. 

Although there is still much to be understood about its pathogenesis, our findings reinforce the definition of reduced brain volume related to atrophy/hypoplasia as one of the possible hallmarks of PKS.

CC anomalies, one of the most common SBAs in congenital syndromes and reported in more than 200 conditions [78], were found to be the second most frequent abnormality in our population, three times more frequent than reported in the literature through any imaging and/or anatomopathological method and twice as frequent as reported through MRI.

The thickness of CC has been associated with cognitive impairment in individuals with different clinical conditions [79] and gait alterations in elderly individuals [80, 81]. In our case series, we found no significant correlations between the thickness of this and the neurodevelopmental delay severity. However, the available neuropsychological data of our population did not allow us to make more accurate correlations, and targeted studies on cognitive profile, motor aspects, and neuroradiological alterations could help in assessing this hypothesis.

Qualitatively, a “shortened” appearance of the CC, with greater thinning at the splenium, was observed. The quantitative analysis confirmed a tendency toward smaller splenium diameters, isolated or sometimes in the context of global hypoplasia. Temporal horn enlargement and atrial widening were variably noted, and they contribute to reinforce the finding of reduced posterior CC commissure. From a neuroanatomical and neurophysiological point of view, the splenium is a key point for the connection of occipital, temporal, and parietal lobes; posterior callosal lesions involving the splenium cause an interhemispheric disconnection between inputs from the left visual field and language processing in the left hemisphere [82, 83]. Auditory processing and comprehension impairment have also been observed in patients with splenial alterations, such as by interrupting the callosal connections of the temporal-parietal junction [84]. Children with PKS have frequent impairment of the visual and auditory pathways. However, visual impairment in our population has not been studied systematically, and the correlation between visual impairment and splenium abnormalities was not significant. Further targeted studies are needed in this regard.

As previously reported, cerebral parenchymal abnormalities were common in our series, accounting for almost one-fifth of the cases. Namely, one individual presented focal cerebral T2-weighted WM hyperintensities reminiscent of those seen in cerebrovascular disorders; he was the oldest patient enrolled in the study (Case 31, 17 years old). Saito et al. described a similar pattern in two older PKS patients. Whether these represent acquired lesions and whether the regression of some motor skills encountered in some patients could mirror the appearance of these lesions during adulthood is still a matter of debate [26]. In 4 out of the 6 PKS individuals with cerebral parenchymal abnormalities, we found lesions evocative of middle cerebral artery stroke and perinatal hypoxic-ischemic injury. Brain clastic lesions have rarely been described in PKS [6, 11, 26, 85], and to our knowledge, there have been no previous PKS cases reporting neuroimaging related to large vessel stroke or perinatal brain vascular hypoperfusion [11]. 

Nearly half of the patients in our population had MCD, almost all with variable degrees of perisylvian PMG. PMG is one of the most common MCDs, accounting for approximately 20% of all MCDs [86], and the perisylvian location accounts for approximately 60–70% of cases, particularly the posterior aspect of the fissure [87]. Several genes have been found to cause PMG, such as WDR62, NDE1, KATNB1, TUBB2B, DYNC1H1, RTTN, and PIK3R2 [88]. Interestingly, CCND2, a gene located at 12p13.32, is also responsible for PMG. Its gain-of-function mutation caused megalocephaly PMG-polydactyly hydrocephalus syndrome (MPPHS3). Hiraiwa et al. speculated that increased dosages of CCND2 could affect neuronal migration in PKS patients, leading to PMG [42, 89]. 

Nongenetic causes of PMG most often include cytomegalovirus (CMV) infection, exposure to teratogens (including alcohol abuse), hypoxic-ischemic insults, or arterial ischemic infarct twin-to-twin transfusion syndrome [87, 90]. 

To date, only six PKS patients with PMG have been described in the literature, and among them, three of six cases showed unilateral or asymmetric PMG [42]. The much higher prevalence of PMG (14 patients, 48%) encountered in our study may lie in the previously mentioned more targeted analysis of brain MRI, as well as in the higher definition of currently available MRI, the majority of which is equipped with 3D T1 sequences. In our cohort, PMG were all perisylvian, most of them bilateral and symmetrical, preferentially involving the deeper part of the fissure. It is worth discussing that between the four unilateral perisylvian PMGs, two individuals presented a contralateral homotopic clastic ischemic lesion, suggesting a possible vascular contribution to the pathogenesis of PMG.

Perisylvian PMG has been previously associated with intellectual disability, impaired (mostly orofacial) motor skills, and epilepsy [88]. All these aspects are present in PKS patients to varying degrees. Izumi et al. suggested that PMG could be an explanation for encephalopathy with focal seizures in PKS [11]; however, we found no statistical correlations between PMG and the presence of epilepsy or the severity of the developmental delay.

Posterior fossa abnormalities were less often encountered in our population and were represented by the enlargement of the cisterna magna and cerebellar vermis malrotation. These data are consistent with previous literature. Interestingly, Greenbaum et al. described a patient with vermis hypoplasia carrying a genetic alteration in arr12p13.3p11.1 [91]. 

Finally, we found a slightly higher prevalence of pineal gland cysts in the studied population than commonly found in pediatric age [92]. The pineal gland regulates circadian rhythm through melatonin production and release, and its function can be impaired due to accidental and developmental conditions, such as pineal tumors, craniopharyngiomas, injuries affecting its sympathetic innervation, and rare congenital disorders. However, pineal cysts in children are benign, incidental findings, for which follow-up is not required if there are no referable symptoms or excessive size [92–95]. Pineal involvement in PKS has been previously reported in a 15-year-old girl who developed a pineal gland tumor, very similar to Case 17 in our population in which a clearly tumoral appearance was present and for which periodic follow-up was set up so far. In that case, the authors supposed a genetic link between the 12p anomaly and tumorigenesis [46], being isochromosome 12p observed in sporadic pineal germ cell tumors [96]. Recently, Izumi et al. described the cooccurrence of PKS and Burkitt lymphoma, strengthening the suspicion of a link between isochromosome 12p and tumorigenesis, with several genes located in the PKS 12p critical region (such as ETV6, KRAS, CCND2, CDKN1B, and AICDA) known to play roles in tumor development [20]. 

Sleep disturbances in PKS patients are common and often severe, and their etiology is multifactorial.^9^ Therefore, the role of these abnormalities in the etiopathogenesis of sleep disturbances is difficult to determine, and should be better investigated through dedicated studies.

### Limits

Due to mosaicism of the chromosomal disorder, the phenotype of PKS is wide and variable; milder cases often go undiagnosed. A main limitation is that our cohort mainly consisted of PKS individuals with a severe phenotype who were referred to the hospital for the presence of epilepsy or other major neurologic or systemic involvement; few individuals with a mild phenotype were included. A future challenge is to be able to extend studies to milder phenotypes.

Moreover, clinical data were collected at different times of medical history in a non-standardized manner. Thus, no conclusive data can be provided in this regard.

Finally, the genetic investigation in our cohort was not homogeneous, therefore preventing a genotype-phenotype correlation.

## Conclusions

SBAs are common in PKS and occur much more frequently than previously reported.

Bilateral perisylvian PMG, described in only 5% of patients in the literature, was a main aspect of our population A certain rate of cerebral volume reduction was confirmed, but it was not possible to discern whether it was age-related and/or worsened with time. Moreover, the appearance of the CC seems distinctive.

PKS is a syndrome whose diagnosis requires specific genetic investigations, sometimes resulting in false negatives. The broadening of knowledge on SBAs in PKS using qualitative and quantitative methods could provide in the future an additional tool for early diagnosis, also laying a foundation for potential future studies on prenatal diagnosis of the syndrome. Moreover, further targeted studies to investigate the possible correlations with both genotype and phenotype may help to determine the prognostic value of these data and increasingly define the etiopathogenesis of the neurologic phenotype of this syndrome.

## Data Availability

The datasets used and/or analyzed during the current study are available at “*IRCCS Istituto delle Scienze Neurologiche di Bologna, UOC di Neuropsichiatria dell’Età Pediatrica”*. The corresponding author can provide them on reasonable request. The complete database, with clinical and demographic data cannot be shared openly to protect study participant privacy according with the policy of our local ethics committee.
